# Changes in Tryptophan Metabolism on Serotonin and Kynurenine Pathways in Patients with Irritable Bowel Syndrome

**DOI:** 10.3390/nu15051262

**Published:** 2023-03-03

**Authors:** Cezary Chojnacki, Aleksandra Błońska, Paulina Konrad, Marcin Chojnacki, Marcin Podogrocki, Tomasz Poplawski

**Affiliations:** 1 Department of Clinical Nutrition and Gastroenterological Diagnostics, Medical University of Lodz, 90-647 Lodz, Poland; 2 Biohazard Prevention Centre, Faculty of Biology and Environmental Protection, University of Lodz, Pomorska 141/143, 90-236 Lodz, Poland; 3 Department of Pharmaceutical Microbiology and Biochemistry, Medical University of Lodz, 92-216 Lodz, Poland

**Keywords:** irritable bowel syndrome, L-tryptophan, serotonin, kynurenine, kynurenic acid, quinolinic acid

## Abstract

(1) Background: L-tryptophan is a substrate for the synthesis of many biological compounds through the serotonin and kynurenine pathways. These compounds have a significant influence on gastrointestinal functions and mental processes. The aim of the study was to evaluate the urinary excretion of selected tryptophan metabolites in patients with constipation-predominant and diarrhoea-predominant irritable bowel syndrome (IBS-C and IBS-D, respectively), related to somatic and mental symptoms. (2) Methods: 120 people were included in the study and three groups were distinguished, with 40 individuals each, including healthy subjects (controls), patients with IBS-C and patients with IBS-D. The Gastrointestinal Symptoms Rating Scale (GSRS-IBS) was used to assess the severity of abdominal symptoms. The Hamilton Anxiety Rating Scale (HAM-A) and Hamilton Depression Rating Scale (HAM-D) were used to evaluate the mental state of patients. Using liquid chromatography tandem mass spectrometry (LC-MS/MS), L-tryptophan and the following metabolites in urine, related to the creatinine level, were measured: 5-hydroxyindoleacetic acid (5-HIAA), kynurenine (KYN), kynurenic acid (KYNA) and quinolinic acid (QA). (3) Results: In both groups of patients with IBS, changes in tryptophan metabolism were found as compared to the control group. We observed an increase in the activity of the serotonin pathway and a positive correlation between the 5-HIAA level and the GSRS score (*p* < 0.01) and HAM-A score (*p* < 0.001) in IBS-D patients. The IBS-C group was characterized by a higher concentration of kynurenines (KYN, QA) in urine. Moreover, the QA (*p* < 0.001) and KYNA (*p* < 0.05) levels were correlated with the HAM-D score among IBS-C patients. (4) Conclusions: Various changes in the tryptophan metabolism pathway can determine the differences in the clinical picture of irritable bowel syndrome. These results should be included in the nutritional and pharmacological treatment of this syndrome.

## 1. Introduction

Irritable bowel syndrome (IBS) is a functional disease of the gastrointestinal tract, characterized by abdominal pain, bloating, flatulence, altered bowel habits and accompanying changes in mental mood [1,2]. The diagnosis is made solely upon the basis of symptoms, after ruling out organic diseases. The different types of IBS are classified according to the predominant stool pattern, which is diagnosed using the Bristol scale [3]. There are four subtypes of IBS, namely, constipation-dominant (IBS-C), diarrhoea-predominant (IBS-D), mixed bowel habits (IBS-M) and unclassified (IBS-U). A hallmark of these symptoms is their variability and recurrence. The pathogenesis of IBS is not clearly understood. Multiple factors may be involved in the development of IBS, including alternation of gastrointestinal motility, visceral hypersensitivity, changes in the gut microbiota, dysfunction in the gut–brain axis and others [4].

Some of these factors are likely to derive from the impairment of the tryptophan (TRP) metabolism pathway underlying the production of the main neurotransmitter of the GI tract—serotonin (SER) [5,6]. SER also has a significant effect on mood, but its level does not always correlate with the clinical symptoms of IBS [7].

TRP is a substrate for the synthesis of many biological active compounds via the SER or kynurenine (KYN) pathways. Nearly 5% of TRP entering the body with food is metabolized through the SER pathway. In the gut, with the involvement of tryptophan hydroxylase (TPH-1), TRP is metabolized to 5-hydroxytryptophan, then to 5-hydroxytryptamine (5-HT, SER) and finally to melatonin (MEL). The final SER metabolite is 5-hydroxyindoleacetic acid (5-HIAA), which serves as an activity marker for the SER pathway [8]. Most of the remaining TRP is metabolized via the KYN pathway, with the participation of indoleamine 2,3-dioxygenase (IDO-1) [8,9]. KYN metabolites, including kynurenine acid (KYNA), can exert neuroprotective effects, while others such as KYN and quinolinic acid (QA) are neurotoxic [10,11,12,13] and can have an impact on the severity of somatic symptoms, as well as mental conditions in human subjects with IBS. In healthy individuals, a physiological balance is maintained between the activity of both pathways. The imbalance between these TRP pathways has been adopted as the basis for the concept of depression and other non-mental illnesses, including IBS [14].

Confirming the roles of tryptophan metabolites in the development of IBS, some studies have identified the modulation of TRP metabolism along the SER and KYN pathways in IBS patients; however, the main focus should be mainly on SER pathway activity [15,16,17,18,19] without considering the possible impact on different forms of IBS and the psychosomatic state of patients with IBS. Furthermore, these studies have focused on and highlighted the influence on tryptophan metabolism, as they presumably affect several key physiological processes and metabolic pathways, thus playing a crucial role in human health. The availability of TRP in the gut is strongly affected by the metabolic activity of the human gut microbiota and consequently also translates into serotonergic signalling modifications in the central nervous system, as TRP mediates communication between the gut and the brain. TRP with the contribution of enzymes produced by the intestinal microbiota leads to the formation of a number of indole products, i.e., 3-indoxyl sulphate and indole-3-propionate (IPA) or indole-3-acetate (IAA). The most straightforward clinical manifestation of the microbial background of IBS is the occurrence of small intestinal bacterial overgrowth (SIBO). It is a hallmark of changes in the gut microbiota and is associated with more than 30% of cases of IBS [20]. However, this does not explain the clinical manifestations of IBS that are not related to microbial dysbiosis.

The aim of our study was to evaluate the urinary excretion of tryptophan metabolites deemed as markers of activity of SER and KYN pathways in patients with two different IBS forms: constipation-predominant and diarrhoea-predominant (IBS-C and IBS-D, respectively), related to somatic and mental symptoms in selected group of IBS patients without SIBO and other signals indicating changes in the bacterial flora.

## 2. Materials and Methods

### 2.1. Patients

The study involved three equal groups of 40 patients: healthy subjects without complaints (controls), 40 patients with IBS-C and 40 patients with IBS-D, aged 23 to 61 years, recruited in the period of 2017–2021. The IBS-C as well as the IBS-D group were classified according to the Rome IV criteria. In the IBS-C group, there were two or fewer bowel movements a week and hard and lumpy stools for a minimum of six months. In the IBS-D group, there were loose or watery stools, which occurred regularly for six months with a minimum of 25% of the time. The number of loose stools ranged from 3 to 8 a day and did not coexist with occasional constipation. In addition, patients complained about abdominal pain related to defecation, bloating and flatulence, and anxiety, depressed mental mood and sleep disturbances.

### 2.2. Diagnostic Procedures

The severity of abdominal symptoms was assessed using The Gastrointestinal Symptom Rating Scale—Irritable Bowel Syndrome (GSRS-IBS) [21]. This scale includes 13 severities of gastrointestinal symptoms occurring within the last seven days of the examination. GSRS-IBS assesses the severity of abdominal pain (1), pain relieved by a bowel action (2), bloating (3), passing gas (4), constipation (5), diarrhoea (6), loose stools (7), hard stools (8), urgent need for bowel movement (9), incomplete bowel emptying (10), fullness shortly after a meal (11), fullness for a long time after eating (12) and visible distension (13). Each item is scored on a 1 to 7 point scale. The GSRS test is completed by the patients after proper appropriate instruction, emphasizing the importance of the answers to achieve the objectives of the study and improve their health.

The breath test was performed using 75 mg of ^13^C-labelled urea and the FAN-ci 2 device set (Fisher Analysen Instrumente, GmbH, Hamburg, Germany). The result was considered to be negative for *H. pylori* infection when the concentration of carbon dioxide in the expired air was below 4.0 ppm percent per million at 30 min. Small intestinal bacterial overgrowth (SIBO) was ruled out on the basis of the results of the lactulose hydrogen breath test using a Gastrolyzer (Bedfont, Ltd., Harrietsham, UK). The result was considered to be negative when an increase in the concentration of hydrogen in breath air was below 20 ppm within 90 min of ingesting 25 mL of lactulose in accordance with the currently accepted criteria.

To determine other diseases of GI tract, all patients underwent endoscopic and histological examination of gastric, duodenal, small intestinal and colonic mucosa, using a standardized method.

At the beginning, patients self-assessed their mental state, and then everyone was evaluated with respect to their mental state using the Hamilton Anxiety Rating Scale (HAM-A) and the Hamilton Depression Rating Scale (HAM-D). European standards were taken for both scales: 10–19 points—mild anxiety/depression, 19–29 points—moderate anxiety/depression, over 30 points—severe anxiety/depression [22].

We excluded participants with H-pylori-induced gastritis, lymphocytic and ulcerative colitis, celiac disease, Crohn disease, allergy and food intolerance, infectious, liver and renal diseases, diabetes, severe anxiety or depression or using of antibiotics, probiotics and psychotropic drugs in the month prior to enrolment into the study.

### 2.3. Laboratory Tests

The following panel of routine tests was conducted for each individual subject: blood cell count, protein quantification, glucose, glycated haemoglobin, profile of lipids, bilirubin, iron, urea, creatinine, thyroid-stimulating hormone, free thyroxine, free triiodothyronine antibodies against tissue transglutaminase and deaminated gliadin peptide, as well as the activity of alanine and asparagine aminotransferase, alkaline phosphatase, gamma-glutamyltranspeptidase, amylase and lipase.

Latex agglutination photometric assay was used to assess the serum concentration of C-reactive protein (CRP) (COBAS INTEGRA 800, Roche Diagnostic, Basel, Switzerland). Faecal calprotectin (FC) was estimated via the ELISA test (Quantum Blue Reader, Buhlmann Diagnostics, Amherst, NH, USA). Urine samples were collected in the morning on an empty stomach into a special container with a 0.1% hydrochloric acid solution as a stabilizer. Liquid chromatography with tandem mass spectrometry (LC–MS) was used to determine TRP and its following metabolites: KYN, KYNA and QA concentration, according to the manufacturer’s instructions (Ganzimmun Diagnostics AG, Mainz, Germany; D-ML-13147-01-01, accepted by the European Parliament—No 765/2008). The levels of these compounds were expressed in mg per gram of creatinine (mg/gCr). The ratios of 5-HIAA and TRP as well as KYN and TRP were also calculated. The 5-HIAA/TRP ratio is considered to be an index of SER pathway activity, while the KYN/TRP ratio is a depiction of KYN pathway activity in TRP metabolism.

### 2.4. Nutritional Intervention

All subjects were ordered to record in a nutrition diary the type and amount of food consumed each day for 14 days into the study. The average daily TRP intake was calculated using a nutrition calculator with the Kcalmar.pro-Premium app (Hermex, Lublin, Poland). The content of TRP and other compounds of a diet in food products were determined in accordance with the findings of the Polish National Institute of Public Health. On the day of the evaluation, everyone was administrated a balanced diet of total caloric value 2000 kcal and with daily intake of a minimum of 50 g protein, 270 g of carbohydrates and 70 g of fats. Products with a rich TRP content, such as wheat, bread, pasta, sweets, hard cheeses, white meat, lean fish and cakes, as well as raw and boiled fruits and vegetables, were limited so that the content of this amino acid in the diet was comparable to previous individual determinations. On the next day, in the morning, the subjects collected urine in a special container, which was immediately sent to the laboratory to determine the level of tryptophan and its metabolites. At the same time, fasting venous blood was collected for routine laboratory tests. The research was conducted as an open-label clinical trial.

### 2.5. Ethical Issues

All subjects were acquainted with the purpose of the study and gave their written consent to perform it. The study was conducted according to the guidelines of the Declaration of Helsinki and the Guidelines for Good Clinical Practice and was approved by the Bioethics Committee of the Medical University of Lodz (RNN/176/18/KE).

### 2.6. Statistical Analysis

Normality of data distribution was checked using the Shapiro–Wilk W test. The Mann–Whitney U test was used to compare the difference between two groups and ANOVA on ranks was used to compare the difference between more than two groups with the Bonferroni–Dunn post hoc test. The correlations between the quantitative variables were analysed using Spearman’s rank test. Data are expressed as mean ± SD if data have a normal distribution, or median and percentiles in other cases; the value *p* < 0.05 was considered to be statistically significant. Sample size was calculated using the Sample Size Calculator within Statistica 13.3 software. α = 0.05 (bilateral) and β = 0.10, and the number of groups was k = 3; the minimum sample size of 34 cases per group was calculated. The internal consistency of questionnaires was calculated using Cronbach’s alpha. The value of overall internal consistency of questionnaires, expressed by Cronbach’s alpha, was around 0.65, which is considered to be acceptable. All statistical analyses were performed using STATISTICA 13.3 software (TIBCO Software Inc., Palo Alto, CA, USA).

## 3. Results

### 3.1. Comparison of Irritable Bowel Syndrome Patients with Controls

All analysed groups had similar values of selected biochemical blood parameter scores. Please note that TRP consumption was also similar in all groups (Table 1).

**Figure 1:**
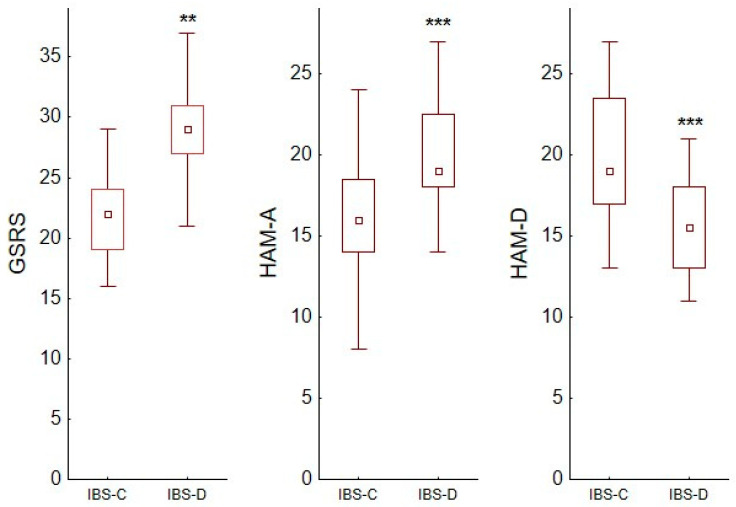
The score (points) of the Hamilton Anxiety Rating Scale (HAM-A) and the Hamilton Depression Rating Scale (HAM-D) in patients with constipation-predominant (IBS-C) and diarrhoea-predominant (IBS-D) irritable bowel syndrome; ** *p* < 0.01; *** *p* < 0.001. Box and whiskers represent median, IQR and min–max value.

The intensity of somatic symptoms (GSRS) in group IBS-C was lower than in IBS-D patients (22.3 ± 3.63 vs. 28.6 ± 3,96; *p* < 0.01). These two groups were also different in terms of the severity of anxiety (16.3 ± 3.66 vs. 19.9 ± 3.19) and depression (20.1 ± 3.89 vs. 15.4 ± 2.70) (Figure 1).

### 3.2. Urinary Excretion of TRP and Its Metabolites

**Figure 2:**
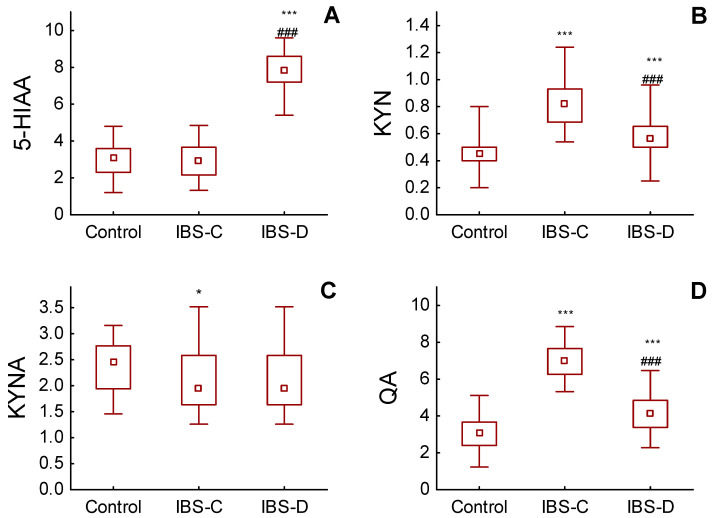
Urinary excretion of (**A**) 5-hydroxyindoleacetic acid (5-HIAA), (**B**) kynurenine (KYN), (**C**) kynurenic acid (KYNA) and (**D**) quinolinic acid (QA) in mg/gCr in healthy subjects (C) and in patients with constipation-predominant (IBS-C) and with diarrhoea-predominant (IBS-D) irritable bowel syndrome (IBS-D); *** *p* < 0.001, * *p* < 0.05, ### *p* < 0.001 between IBS-C and IBS-D. Box and whiskers represent median, IQR and min–max value.

The excretion of TRP in urine did not differ between groups and was consistent (Table 2 and Figure 2).

**Figure 3:**
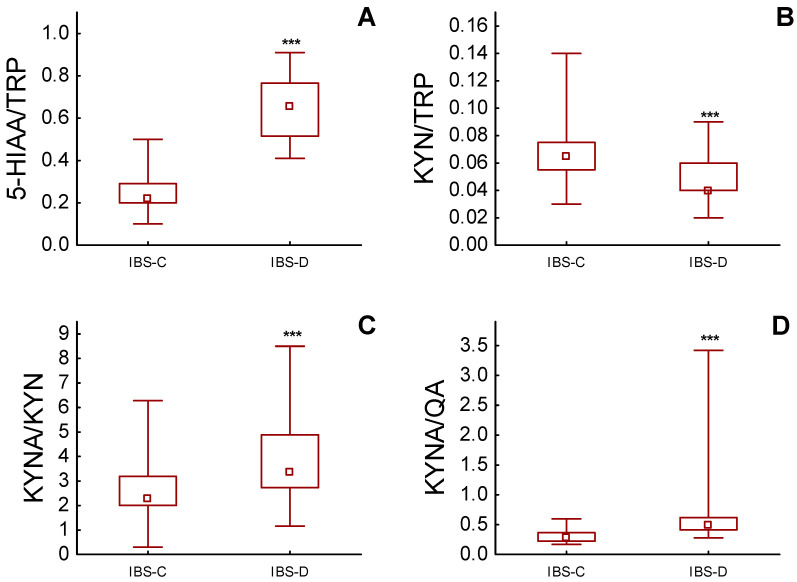
Ratios between urinary levels of (**A**) 5-hydoxyindoleacetic acid (5-HIAA) and tryptophan (TRP), (**B**) kynurenine (KYN) and TRP, (**C**) kynurenic acid (KYNA) and KYN and (**D**) kynurenic acid and quinolinic acid (QA) in patients with constipation-predominant (IBS-C) and diarrhoea-predominant (IBS-D) irritable bowel syndrome; *** *p* < 0.001. Box and whiskers represent median, IQR and min–max value.

We also calculated the ratio between urinary levels of 5-HIAA/TRP, KYN/TRP, KYNA/KYN and KYNA/QA and compared them between IBS-C and IBS-D groups. In all of these instances, the calculated ratios were higher in IBS-D patients (Figure 3A–D).

In IBS-C patients, a positive correlation between QA levels and the HAM-D score (*p* < 0.001), and a negative correlation between KYNA levels and the HAM-D score (*p* < 0.05), were found. In IBS-D patients, a positive correlation between 5-HIAA levels and GSRS score (*p* < 0.01) and HAM-A score (*p* < 0.001) was found (Table 3).

## 4. Discussion

The results obtained in this work confirm the participation of tryptophan and serotonin in the pathogenesis of IBS without SIBO. Research on this issue has been ongoing for a long time, but the results have been inconclusive. All previous studies have focused mainly on changes in serotonin homeostasis, but the conclusions have been varied. Beacroft et al. showed higher postprandial serum serotonin levels in patients with IBD-D [23]. Houghton et al. also found high serotonin secretion, especially in women with IBS–D [24]. Moskwa et al. found increases in serum levels in patients with IBS-D and IBS-C [25]. Dunlop et al. found a decrease in serotonin secretion in IBS-C patients and an increase in patients with serum levels in post-infectious IBS [26]. Atkinson et al. observed a high fasting and postprandial concentration of serotonin in patients with IBS-D and no changes in IBS-C patients [27]. Similarly, Spiller et al. found that IBS-D is associated with an increased postprandial level of SER, while IBS-C is associated with an impaired SER response [28]. El-Salhy et al. found a reduced number of EC cells in both subtypes of IBS [29]. Coates et al. in both types of IBS found lower-level expression of TPH-1 and SERT in the colonic mucosa, but an unchanged number of CEs [30]. Miwa et al. found a significant increase in SER concentration in the colonic mucosa in IBS-C, while the urinary excretion of 5-HIAA was similar in both types of IBS [31]. Yu et al. showed in IBS-D patients a higher blood concentration of SER and 5-HIAA and an increased expression of TPH-1 in the colonic mucosa, similar to patients with ulcerative colitis [32]. In our subsequent research in patients with IBS-D [33] and lymphocytic colitis [34], similar results were obtained. Thijssen et al. demonstrated that fasting SER plasma levels were not significantly different in all subtypes of patients with IBS, but 5-HIAA levels and the 5-HIAA/SER ratio decreased. Furthermore, no correlations were found between serotonin signalling data and somatic and psychological symptoms [35].

All of the above results show the participation of SER in the pathogenesis of IBS, but they are not coherent. However, they do not explain the causes of comorbid mood disorders. In recent years, the focus has been more on tryptophan metabolism via the kynurenine pathway, but the obtained results have also not been conclusive. Both the serotonin and kynurenine pathways compete for the substrate (TRP). Increased consumption of TRP by the kynurenine pathway can result in too little TRP and SER in both the CNS and GIT. The imbalance of these pathways may explain the variability and recurrence of IBS symptoms. Attempts were made to confirm these suggestions in further research. Clarke et al. observed an increase in plasma KYN levels and the KYN/TRP ratio in patients with IBS compared to healthy people. The authors interpreted their results as being the consequence of an increase in the activity of IDO, an enzyme responsible for TRP degradation. However, it must be noted that the study was performed in only nine male patients, and the type of disease was not specified [36]. Heitkemper et al. found a lower KYN/TRP ratio in 38 women with IBS-D compared to healthy people [37]. Kaszthelyi et al. showed that IBS patients had a higher blood concentration of SER and KYNA compared to healthy controls [38]. Fitzgerald et al. indicated that IBS patients had an increased plasma KYN concentration and KYN/TRP ratio compared to healthy subjects. Furthermore, the KYN/TRP ratio was positively correlated with the severity of abdominal complaints, as well as with anxiety and depressive syndrome [39]. However, Christmas et al. [19] in patients with IBS-D showed significantly free serum tryptophan and significantly lower tryptophan dioxygenase. The authors concluded that in IBS-D patients, tryptophan metabolism along the kynurenine pathway is inhibited and these findings are consistent with possible increased SER activity. Increased levels of serotonin have been found to be responsible for increased intestinal motility and diarrhoea.

The results of the above studies were mainly determined in plasma or serum. In our investigation, these compounds were determined in urine, making it difficult to compare the results. These metabolites do not accumulate in the body and come from the transformation of TRP in both peripheral organs and CSN. Furthermore, such determinations provide insight into the many hours of tryptophan metabolic processes. The results obtained do not confirm the inverse relationship between activity and both pathways of tryptophan metabolism, but they do not exclude the competition of the appropriate enzymes in the access to TRP. However, they conclude that in IBS patients there is greater activity of the serotonin pathway compared to IBS-C patients. In IBS-C patients, the activity of the kynurenine pathway was greater than in IBS-D patients. However, in the IBS-C group, a higher KYN/TRP ratio was found, while lower KYNA/KYN and KYNA/QA ratios were found. These changes indicate the advantage of neurotoxic compounds over neuroprotective ones. We previously found similar changes in people with depression and the tract of the small intestinal bacterial overgrowth [40]. In patients, a positive correlation was found between 5-HIAA excretion and abdominal complaints and anxiety. However, in patients with IBS-C, there was a positive correlation between levels and depressive symptoms, and a negative correlation between KYNA levels and the HAM-D score. From the above, it follows that changes in tryptophan metabolism in both the serotonin and kynurenine pathways may be the cause of somatic and mental ailments in patients with IBS.

Our results do not exclude the participation of other factors in the pathogenesis of IBS. In patients with IBS, somatic symptoms can worsen under psychological stress [41]. On the contrary, chronic abdominal complaints cause anxiety and worsen mood in these patients [42]. Thus, the vicious circle of psychosomatic disorders is closed. For this reason, in patients with IBS, antidepressants are used. Their use is limited by side effects. Drugs that modulate the activity of enzymes in tryptophan metabolism are still being sought. However, a proper diet with the restriction of tryptophan intake or its supplementation may be an important condition for the effective treatment of this disease [43,44], which should be considered in practice. The test of tryptophan metabolites should be especially performed in patients with coexisting mood disorders and suboptimal TRP intake. There is an important influence on somatic and mental symptoms in IBS patients with intestinal bacteria. Under the influence of many factors, including not eating a proper diet and chronic stress, there are changes in gut microbiota. The excessive growth of these bacteria, even nonpathogenic species, causes ailments, often similar to irritable bowel syndrome. This coincidence leads to the recognition of the bacterial theory of the pathogenesis of IBS. Many studies have shown the impact of the microbiome on the central nervous system and the existence of a communication pathway between the gut microbiota and the brain. The mechanism of these dependences is not fully understood. It is assumed that some biological compounds, including serotonin, send signals through the vagus nerve to the brain, with information about changes in the gut microbiome. In turn, signals sent from the brain affect the state of the gut microbiome [45]. Some species of these bacteria, by increasing the production of neurotransmitters, can increase the stresses on the function of the nervous system [46].

Our study has some limitations. The negative results of the hydrogen breath test do not rule out other changes in gut microbiota, whose diagnosis is still imperfect. The results of the LHBT test only relate to the quantitative composition of bacteria in the small intestine. Most bacteria colonize the large intestine and have a significant impact on the functions of this organ in functional diseases. For this reason, further methods of testing the intestinal microbiota are necessary. A future promise to overcome the deadlock related to this may be a test using IPA urinary analysis. As it would appear, IPA synthesis is preceded by tryptophanase-mediated indole formation, which allows quantitative evaluation in human urine of microbiota-modulated TRP metabolism [47]. IPA was reported to be inversely associated with inflammatory bowel disease [48]. Several other strains of bacteria capable of metabolizing TRP in the serotonin and kynurenine pathways, as well as this, may directly convert TRP into indole and scatols. These metabolites are suggested to activate the immune system by binding to aryl carbon receptors (AHRs), stimulating gastrointestinal motility and the secretion of intestinal hormones.

The research was conducted as an open-label clinical trial. The results of the GSRS test and HAM-A test were based on patients’ responses regarding objective and subjective symptoms, but constant contact with the doctor and dietician ensured their credibility.

## 5. Conclusions

Various changes in the serotonin and kynurenine pathways of tryptophan metabolism may determine the differences in the clinical picture of irritable bowel syndrome. These results should be included in the nutritional and pharmacological treatment of this syndrome.

## Figures and Tables

**Table 1:** General characteristics and the selected biochemical blood parameters in healthy subjects (controls) and in patients with IBS-C as well as IBS-D; data are presented as average ± SD.

Feature	Controls (*n* = 40)	IBS-C (*n* = 40)	IBS-D (*n* = 40)
Age (years)	42.9 ± 10.2	44.5 ± 10.9	43.7 ± 12.3
Gender M/F	8/32	9/31	10/30
BMI (kg/m^2^)	23.2 ± 1.2	24.4 ± 2.3	23.2 ± 3.1
GFR (mL/min)	99.2 ± 4.3	92.2 ± 6.9	96.5 ± 4.8
ALT (µ/L)	14.5 ± 2.8	17.8 ± 3.6	16.1 ± 4.2
AST (µ/L)	12.9 ± 1.9	13.2 ± 2.4	14.5 ± 2.5
CRP (mg/L)	2.54 ± 0.8	3.1 ± 1.7	4.2 ± 1.9
FC (µg/g)	19.8 ± 8.6	28,6 ± 18.8	31.9 ± 18.9
TRP (mg daily)	1329 ± 178	1262 ± 139	1286 ± 155

GFR—glomerular filtrating ratio, ALT—alanine aminotransferase, AST—aspartate aminotransferase, CRP—C-reactive protein, FC—faecal calprotectin, TRP—tryptophan intake; data are presented as average ± SD; differences between groups are not significant.

**Table 2:** Urinary excretion of tryptophan and its metabolites in healthy subjects (controls, C) and in patients with constipation-predominant (IBS-C) and with diarrhoea-predominant (IBS-D) irritable bowel syndrome; median (*M*) ± interquartile range (IQR).

Feature	Controls			IBS-C			IBS-D		
	*M*	IQR		*M*	IQR		*M*	IQR	
TRP (mg/gCr)	12.70	10.90	14.85	12.25	10.10	14.20	12.25	10.10	14.20
5HIAA (mg/gCr)	3.10	2.30	3.60	2.98	2.16	3.67	7.85	7.20	8.60
5-HIAA/TRP	0.22	0.17	0.28	0.23	0.20	0.29	0.66	0.52	0.77
KYN (mg/gCr)	0.45	0.40	0.50	0.82	0.69	0.93	0.57	0.50	0.66
KYN/TRP	0.03	0.03	0.04	0.07	0.06	0.08	0.04	0.04	0.06
KYNA (mg/gCr)	2.45	1.94	2.77	1.94	1.64	2.59	2.01	1.64	2.61
KYNA/KYN	5.93	3.96	6.57	2.31	2.01	3.19	3.44	2.76	4.98
QA (mg/gCr)	3.13	2.40	3.67	6.98	6.26	7.67	4.15	3.38	4.86
KYNA/QA	0.79	0.62	1.06	0.28	0.23	0.37	0.53	0.40	0.62

We observed elevated concentrations of 5-HIAA (*p* < 0.001), KYN (*p* < 0.001) and QA (*p* = 0.011) in the urine of IBS-D patients in comparison to controls (Table 2 and Figure 2). KYN and QA were also elevated in IBS-C patients as compared to controls; however, KYNA was lowered (*p* = 0.041) and 5-HIAA did not differ from the control group and was significantly lower than in the IBS-D group. We also observed a difference between the urinary level of KYN (*p* < 0.001) and QA (*p* < 0.001) between the IBS-C and IBS-D groups.

**Table 3:** Correlation between levels (mg/gCr) of 5-hydoxyindoleacetic acid (5-HIAA), kynurenine (KYN), kynurenic acid (KYNA) and quinolinic acid (QA) and scores of the Gastrointestinal Symptom Rating Scale (GSRS), the Hamilton Anxiety Rating Scale (HAM-A) and The Hamilton Depression Rating Scale (HAM-D) in those with IBS-C and IBS-D. The results are expressed as the Spearman’s rank correlation coefficient (rho).

Tryptophan Metabolites	GSRS		HAM-A		HAM-D	
	IBS-C	IBS-D	IBS-C	IBS-D	IBS-C	IBS-D
5-HIAA	0.029	0.4498 **	−0.0344	0.5064 ***	0.2016	0.2545
KYN	0.0582	0.1829	−0.1115	−0.1706	0.0518	−0.0525
KYNA	0.1526	−0.2314	0.2004	0.1378	−0.3609 *	0.0771
QA	0.0328	0.0081	−0.0326	0.038	0.8200 ***	0.1976

* *p* < 0.05, ** *p* < 0.01, *** *p* < 0.001.

## Data Availability

Data supporting the reported results can be found in C.C. lab upon reasonable request.
